# O-GlcNAcylation of UGDH: emerging insights into immunometabolic regulation and therapeutic implications

**DOI:** 10.1038/s41420-026-03086-y

**Published:** 2026-03-31

**Authors:** Qiang Wu, Ting Lei, Shujing Wang

**Affiliations:** 1 The Department of Thoracic Surgery, The Second Hospital of Dalian Medical University, Dalian, China; 2 Liaoning Provincial Key Laboratory of Glycobiology and Glycoengineering, Dalian Medical University, Dalian, China; 3 Department of Biochemistry and Molecular Biology, College of Basic Medical Sciences, Dalian Medical University, Dalian, China

**Keywords:** Cancer metabolism, Cancer genetics

The study by Lin et al. elucidates a novel mechanism by which O-GlcNAcylation of uridine diphosphate glucose dehydrogenase (UGDH) at serine 350 enhances enzymatic activity, promoting hyaluronic acid (HA) synthesis and extracellular matrix (ECM) remodeling, thereby facilitating tumor immune evasion via reduced CD8^+^ T cell infiltration [1]. While this work provides significant insights into post-translational modification (PTM)-mediated immunometabolic regulation, several aspects warrant further discussion from both conceptual and translational perspectives.

## Novelty and mechanistic insights

The identification of UGDH O-GlcNAcylation as a critical regulator of tumor immunity represents a substantial advancement. Unlike previous studies focusing on O-GlcNAcylation in signaling pathways (e.g., HGFR/PD-L1 degradation [2]), this work directly links metabolic enzyme modification to ECM-driven immunosuppression. The authors demonstrate that O-GlcNAcylation stabilizes UDP-glucose binding through hydrogen-bond reinforcement, increasing enzymatic activity by 8-fold—a finding supported by extensive molecular dynamics simulations [1]. This mechanistic detail underscores the role of PTMs in fine-tuning metabolic flux within the tumor microenvironment (TME), bridging glycosylation with immunoregulation.

However, the generality of this mechanism across cancers remains unexplored. While Lin et al. confirm observations in non-small cell lung cancer (NSCLC), pancreatic, and liver cancer models, UGDH expression varies widely across malignancies. Notably, this mechanism may not be universal. For instance, in glioblastoma, UGDH overexpression correlates with immune activation rather than suppression [3]. This discrepancy suggests context-dependent functions, potentially influenced by tissue-specific ECM composition or immune cell profiles. Thus, the proposed axis (O-GlcNAcylation-UGDH-HA-CD8^+^ T cell exclusion) may not be universally applicable, necessitating validation in immunologically “cold” tumors like prostate or ovarian cancers.

## Immunotherapeutic implications

The study highlights UGDH O-GlcNAcylation as a potential target to enhance immune checkpoint blockade (ICB). By suppressing CXCL10 via KPNA2-STAT1 competition, O-GlcNAcylation creates a chemokine-deficient TME, reminiscent of KRAS-driven cancers where CXCL3/MDSC recruitment impedes ICB efficacy [4]. Targeting O-GlcNAcylation with OGT inhibitors (e.g., OSMI-4) could thus reverse CD8^+^ T cell exclusion, analogous to HA degradation improving ICB response in colorectal cancer [5].

Yet, translational challenges abound. First, global OGT inhibition may disrupt homeostasis in normal tissues, given O-GlcNAcylation’s role in cellular stress response, as it regulates cancer cell metabolic reprogramming and survival stress signaling via HIF-1α–GLUT1 axis to promote oncogenesis [6, 7]. Second, the development of potent and selective OGT inhibitors is hampered by a core obstacle: the enzyme’s highly conserved active site, together with its distinctive structural characteristics, presents major challenges to achieving satisfactory selectivity and drug-like properties [8]. Third, HA accumulation alone may not suffice to suppress immunity; collagen density and fibroblast activation are equally critical [9]. Combining OGT inhibitors with ECM-modulating agents (e.g., collagenase or FAK inhibitors) could synergize but requires careful toxicity profiling. Alternatively, peptide-based strategies targeting UGDH’s glycosylation site might achieve specificity, though delivery to tumors remains obstacles.

To fully explore its clinical translational potential, developing robust, site-specific methods for quantifying UGDH O-GlcNAcylation in patient samples is imperative. A streamlined workflow—enriching target glycopeptides via bioorthogonal labeling or boronate affinity chromatography, separating them with HILIC columns to resolve isomers, and quantifying through targeted mass spectrometry (MS) approaches like parallel reaction monitoring (PRM) and multiple reaction monitoring (MRM-MS)—suits this need well [10]. If validated as a clinically relevant predictive biomarker, UGDH O-GlcNAcylation could facilitate the stratification of patients with “HA-high/CXCL10-low” tumors, identifying those most likely to benefit from combination therapies, such as ICB, hyaluronan (HA)-targeting agents (e.g., PEGPH20), or emerging selective O-GlcNAc transferase (OGT) inhibitors, thereby advancing personalized cancer treatment.

## Controversies and unanswered questions

A key controversy lies in the primary driver of immune exclusion: HA accumulation versus CXCL10 suppression. Lin et al. [1] argue both contribute, but their relative importance is unclear. In pancreatic cancer, HA-rich stroma primarily limits T cell penetration [11], whereas in melanoma, CCL5 deficiency alone suffices to reduce infiltration [12]. Tissue-specific nuances likely exist, and dual targeting (e.g., 4-MU + CXCR3 agonizts) could be explored.

Moreover, the study overlooks crosstalk between O-GlcNAcylation and other PTMs. For instance, phosphorylation near Ser350 could antagonize glycosylation, as seen in other enzymes [13]. Similarly, crosstalk with ubiquitination might modulate UGDH stability [14]. These interactions could explain residual glycosylation in S350A mutants and warrant investigation.

Finally, the clinical relevance of UGDH O-GlcNAcylation levels needs validation. While Lin et al. show correlation with poor prognosis in NSCLC, larger cohorts must assess its predictive value for ICB response. Technologies like mass cytometry-based phospho/glyco-proteomics could multiplex UGDH modification status with immune markers to stratify patients [15].

## Future directions

Prospective studies should: (1) Develop isoform-specific OGT inhibitors to minimize off-target effects; (2) Explore UGDH O-GlcNAcylation in conjunction with other ECM components (e.g., fibronectin or proteoglycans); (3) Investigate temporal dynamics of glycosylation during therapy to identify optimal intervention windows. Integrating single-cell RNA sequencing with spatial transcriptomics could reveal how UGDH-driven ECM remodeling spatially restricts T cells.

In conclusion, Lin et al. provide a foundational framework for targeting immunometabolic pathways via PTMs. Their work underscores the need for multidimensional approaches to overcome immune resistance, positioning UGDH O-GlcNAcylation as a promising, albeit complex, therapeutic node.
